# Study on the Relations between Hyperspectral Images of Bananas (*Musa* spp.) from Different Countries, Their Compositional Traits and Growing Conditions

**DOI:** 10.3390/s20205793

**Published:** 2020-10-13

**Authors:** Zhijun Wang, Sara Wilhelmina Erasmus, Xiaotong Liu, Saskia M. van Ruth

**Affiliations:** 1 Food Quality and Design Group, Wageningen University and Research, P.O. Box 17, 6700 AA Wageningen, The Netherlands; zhijun.wang@wur.nl (Z.W.); sara.erasmus@wur.nl (S.W.E.); xiaotongliu_food@163.com (X.L.); 2 Wageningen Food Safety Research, Wageningen University and Research, P.O. Box 230, 6700 AE Wageningen, The Netherlands

**Keywords:** correlation analysis, geographical origin, organic, VIS-NIR hyperspectral fingerprints

## Abstract

Bananas are some of the most popular fruits around the world. However, there is limited research that explores hyperspectral imaging of bananas and its relationship with the chemical composition and growing conditions. In the study, the relations that exist between the visible near-infrared hyperspectral reflectance imaging data in the 400–1000 nm range of the bananas collected from different countries, the compositional traits and local growing conditions (altitude, temperature and rainfall) and production management (organic/conventional) were explored. The main compositional traits included moisture, starch, dietary fibre, protein, carotene content and the CIE L*a*b* colour values were also determined. The principal component analysis showed the preliminary separation of bananas from different geographical origins and production systems. The compositional and spectral data revealed positively and negatively moderate correlations (r around ±0.50, *p* < 0.05) between the carotene, starch content, and colour values (a*, b*) on the one hand and the wavelength ranges 405–525 nm, 615–645 nm, 885–985 nm on the other hand. Since the variation in composition and colour values were related to rainfall and temperature, the spectral information is likely also influenced by the growing conditions. The results could be useful to the industry for the improvement of banana quality and traceability.

## 1. Introduction

It has been shown that eating bananas provides health benefits in respect of hypertension, cancer, diabetes and depression [1], given their composition of several essential nutrients, such as potassium, vitamins, carotenoids, manganese, fibre and dopamine [2]. Bananas can also be easily added to a regular diet by simply eating fresh fruits or by adding them to other foods (i.e., yoghurt or smoothies). Therefore, bananas are globally frequently consumed fresh fruit. The health benefits associated with the consumption of bananas and related processed products are highly correlated with the essential nutritional contents [3,4], while the quality and composition of bananas are affected by their origin and related growing conditions.

Previous studies have demonstrated the effect of origin and related growing conditions on the composition of fruits. For example, Margraf et al. [5] reported that the geographical origin can influence the total soluble solids of Brazilian grape juices, while research on olive fruits from five different Turkish regions showed that environmental factors can potentially influence the maturity of the fruits and their phenolic fractions [6]. Different geographical parameters, including annual average temperature, altitude and precipitation, can affect the physicochemical properties of fruits. For instance, the polyphenol content of pomegranate juice is highly influenced by temperature in the maturity period and growing latitude [7]. In addition, constant rising temperatures reduce the freshness and change the ruby colour of Bordeaux wines (produced from grapes) by changing the content and metabolism of flavonoids [8]. For bananas, there are only a few papers that have reported the effects of growing conditions on their composition [9,10].

Banana export is one of the important economic pillars for many tropical and subtropical countries, such as the Philippines and Ecuador [11]; banana exports reached a record high of 19.2 million tonnes in 2018. Therefore, traceability of the geographical origin of bananas is quite important for protecting the integrity of the banana supply chain and helps to prevent unfair competition – especially in cases where a premium price is linked to a certain production system or origin. Hence, considering the popularity, health benefits and economic importance of bananas, exploring the relationship between banana quality and growing conditions is necessary. A way to address banana geographical traceability is through data analysis and discrimination methods.

In the last few decades, important technologies have been identified that can be successfully applied to food authenticity and traceability research, such as liquid chromatography–mass spectrometry (LC-MS), nuclear magnetic resonance spectroscopy (NMR), near-infrared (NIR) spectroscopy and hyperspectral imaging (HSI) [12]. HSI coupled with chemometrics has been widely used for studies on food quality and safety [13]. In comparison with other spectroscopy techniques, spectral characteristics as well as spatial information can simultaneously be recorded in one-time scanning with a HSI camera [14]. HSI technology has recently gained wide recognition in the field of food safety. Several studies in the food fraud domain have been conducted using HSI because of its advantages, such as rapidness, accuracy, reproduction and portability [15]. Recently, using HSI, Acierno et al. [16] reported that cocoa bean samples from South America and Africa show reflectance differences in the NIR wavelength range. Sun et al. [17] claimed that the HSI system can provide rapid rice origin identification with a model accuracy of about 92% from combined spectral, morphological and textural features. HSI can reflect the correlation between food composition and the optimum spectrum with the help of chemometrics [18]. On the other hand, food composition is usually influenced by local growing conditions, such as climate, rainfall, organic or conventional production [19]. However, no research has been conducted using HSI to explore its relationship with growing conditions and the physicochemical composition of bananas.

Hence, this study aims to elucidate the relationships between hyperspectral imaging data of bananas (*Musa* spp.) from different countries (Dominican Republic, Ecuador, Colombia, Costa Rica, Panama and Peru) using a portable HSI system in the visible (VIS) and NIR range (400–1000 nm) and compositional traits and the bananas’ growing conditions. The underlying factors were examined by correlation of the spectral data with compositional data of the bananas (physicochemical properties: carotene, starch, water, protein content and colour values) and their growing conditions (altitude, temperature, rainfall and production system). The relationship between datasets is established using chemometrics. The importance of this research relates to bridging the knowledge gap between the interaction of the environment with a food product, and how these extrinsic/environmental factors can influence the intrinsic quality characteristics. It also helps to establish a potential tool by which these characteristics can be measured, and offers a way to trace the geographical origin of a food product.

## 2. Materials and Methods

### 2.1. Sample Collection and Preparation

**Figure 1:**
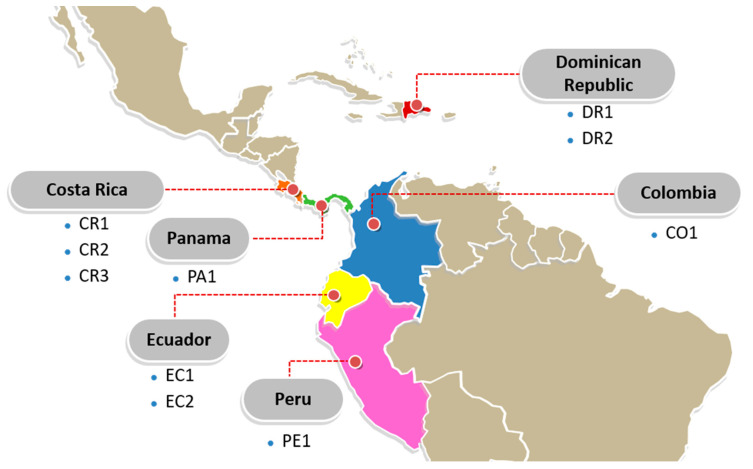
The sampling sites of bananas. (CO: Colombia; CR: Costa Rica; DR: Dominican Republic; EC: Ecuador; PA: Panama; PE: Peru; numbers refer to individual farms in a country). (Map modified based on YourFreeTemplates.com [20]; Shared according to CC BY-ND 4.0).

As shown in Figure 1, the research area was distributed in the main banana-producing areas from 19°42′3.0″ N to 4°51′47.3″ S and from 83°56′27.21″ W to 71°02′12.2″ W. Ten farms were selected as sampling sites; they are located in Central and South America, including Dominican Republic (two farms: DR1 and DR2), Ecuador (two farms: EC1 and EC2), Colombia (one farm: CO1), Costa Rica (three farms: CR1, CR2 and CR3), Panama (one farm: PA1) and Peru (one farm: PE1). Although these regions are known for high temperature and abundant precipitation, differences in the growing condition of bananas still remain due to altitude, latitude, annual precipitation, average temperature and the amount of daily sunshine. To limit external variation and ensure that the only variation came from the growing condition, bananas of the same variety, namely *Cavendish Williams*, were collected in their early maturity phenological stage in the same harvest year of 2018. Ten banana bunches were randomly selected from each farm. Only the bananas from the top position of bunches were selected to obtain high-quality samples. The banana bunches were packaged in pre-cleaned polyethylene bags and transported to Wageningen University and Research by courier in a low-temperature environment (11–13 °C). (Table 1). Two banana fingers from each bunch (per farm) were selected randomly to represent one sample of the specific farm. Ten banana samples were collected from each farm; totaling 100 samples for the study. The peel and pulp of the bananas were separated, freeze-dried and pulverized into a fine powder prior to the HSI image acquisition (Section 2.3) and compositional analysis (Section 2.4 and Section 2.5).

### 2.2. Geographical Data

The Global Positioning System (GPS) coordinates of the various sampling sites were provided by the local farms. The growing condition of bananas, including altitude according to GPS, monthly mean temperature and annual rainfall of harvest year were collected using Google Earth and public databases CRU TS4.04 (Climatic Research Unit (CRU) Time-Series (TS) version 4.04 of high-resolution gridded data of month-by-month variation in climate) [21] (Table 1).

### 2.3. Hyperspectral Imaging

**Figure 2:**
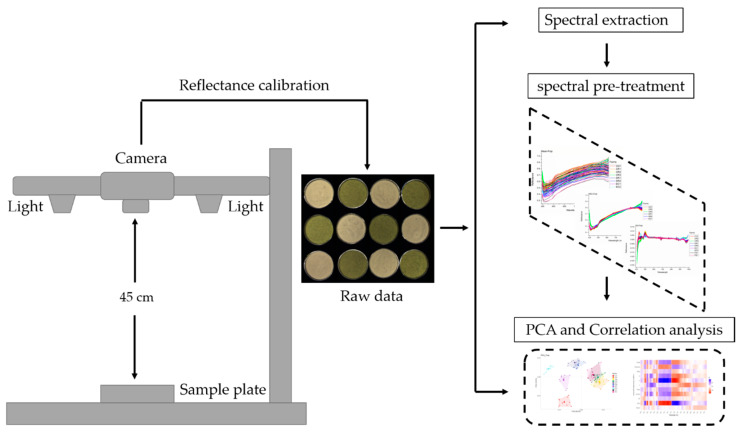
The schematic diagram of the hyperspectral system setup and steps for analysing hyperspectral images. (Please refer to the web version of this article for the interpretation of the colours used in the figure).

The schematic diagram of the hyperspectral system set is shown in Figure 2. The hyperspectral images of banana pulp and peel samples were acquired using a Specim IQ hyperspectral system (Specim, Spectral Imaging Ltd., Finland) coupled with six 50 Watt halogen lamps (Philips, The Netherlands). The Specim IQ is a portable hyperspectral camera that consists of a spectral camera (CMOS technology), a viewfinder camera (5 Mpix), a focus camera and a scanner with a motor for optics movement. The focal length of the camera is 21 mm and the effective resolution of the CMOS camera is 512 × 512 pixels. Banana pulp and peel samples were placed into a plastic container (20 × 30 cm) and then individually placed on a black plate. The distance between the samples and the camera was set to 45 cm. A white Teflon panel was initially captured and used as a white reference prior to the actual measurement. Individual sample images were acquired within 10 ms integration time in a spectral range of 400–1000 nm at 7 nm resolution in line-scan mode [22]. After the full image was scanned, raw data were recorded as separated data and corrected automatically by white and black references by the camera. The subsequent spectral extraction and multivariate data analysis were conducted by ENVI image analysis software (Harris Geospatial Solutions Inc., Broomfield, CO, USA) and MATLAB R2018b (The MathWorks Inc., Torrance, CA, USA) [23].

### 2.4. Determination of Moisture, Starch, Total Dietary Fibre, Protein and Carotene Contents

The moisture content of fresh banana pulp and peel samples were calculated by recording the weight difference before and after freeze-drying, which is expressed as g/100g (wet weight). The following compositions were expressed based on dry weight. Total starch, diet fibre contents of banana pulp and peel were determined by the Total Starch Assay Kit (AA/AMG) and Total Dietary Fibre Assay Kit (Megazyme Ltd., Ireland) [24]. The protein contents were determined by Flash EA 1112 Protein analyser (Thermo Fisher Scientific, USA) using the Dumas combustion method [25]. The carotene content was determined using high-performance liquid chromatography (HPLC) method [26]. For this HPLC method, the carotenes were extracted from the banana pulp and peel samples three times using a hexane and tetrahydrofuran solution until the pellet appeared colourless after centrifugation. The supernatant was then collected and evaporated using a vacuum evaporator. Finally, the extracted carotenes were dissolved in a sample buffer (MeOH–THF 1:1 + 0.01% BHT) and injected into the HPLC with a UV detector at 245.0 nm on an Agilent 1200 Infinity chromatograph. The mobile phase consisted of acetonitrile (A), methanol (B), ethyl acetate (C) at ratios of 60:30:10 (*v:v:v*) and 0.1% triethylamine. With 20 μL injection volume, samples were separated on a Phenomenex Geminin C18 Column (5 μm, 250 mm × 4.6 mm) at 30 °C with a flow rate of 1.0 mL/min. A series of standard solutions were prepared by β-carotene (Sigma-Aldrich, USA). Each sample was prepared in triplicate.

### 2.5. Colour (L*a*b*) Value Measurements

Colour values of the banana pulp and peel samples were determined using a Color Flex spectrophotometer (Hunter Associates Laboratory, Inc., Reston, USA) [27]. The colour meter was first standardised using a green tile, after which the banana pulp and peel powder samples were weighed (2 mg) into cuvettes, and the L*, a* and b* values measured. Each sample was measured three times to obtain an average colour reading.

### 2.6. Statistical Analysis

#### 2.6.1. Data Processing of Hyperspectral Images

As shown in Figure 2, after acquiring the images, the region of interest (ROI) was selected using the ENVI 5.3 software (Harris Geospatial Solutions Inc, America.), and the full-pixel spectra of all banana pulp and peel samples were extracted in MATLAB R2018b. The spectral value of the average spectrum of each sample was collected to generate discrimination models of geographical origin. Prior to the development of the models, spectral pre-treatment of the raw data was performed using the multiplicative scatter correction (MSC) and Savitzky–Golay (SG) methods to reduce background noise and improve spectral resolution [28]. After the spectral pre-treatment, principal component analysis (PCA) was performed for multivariate exploration of image data. Kendall correlation coefficients (r) for the spectral data, chemical composition, colour values and the growing conditions of bananas were generated in a heatmap to show their relationships by R 3.5.3 software (R Foundation for Statistical Computing, Vienna, Austria).

#### 2.6.2. Data Analysis of Banana Compositions

The Shapiro–Wilk test was conducted to determine if the data was normally distributed [29]. Then, all the compositional data were further analysed and reported as mean values ± standard deviation. The significant level was determined by one-way analysis of variance (ANOVA) with Tukey’s significant difference at a 5% significance level (*p* < 0.05). The relationship between chemical composition and growing conditions of bananas were evaluated by Kendall’s correlation coefficients (r). All statistical analyses were performed using Unscrambler 10.5 (Camo Analytics, Norway) and R 3.5.3 (R Foundation for Statistical Computing, Austria) software.

## 3. Results and Discussion

### 3.1. Explorative Analysis of the Spectral Features, Compositional Traits of Banana Pulp, Peel and Related Growing Conditions

**Figure 3:**
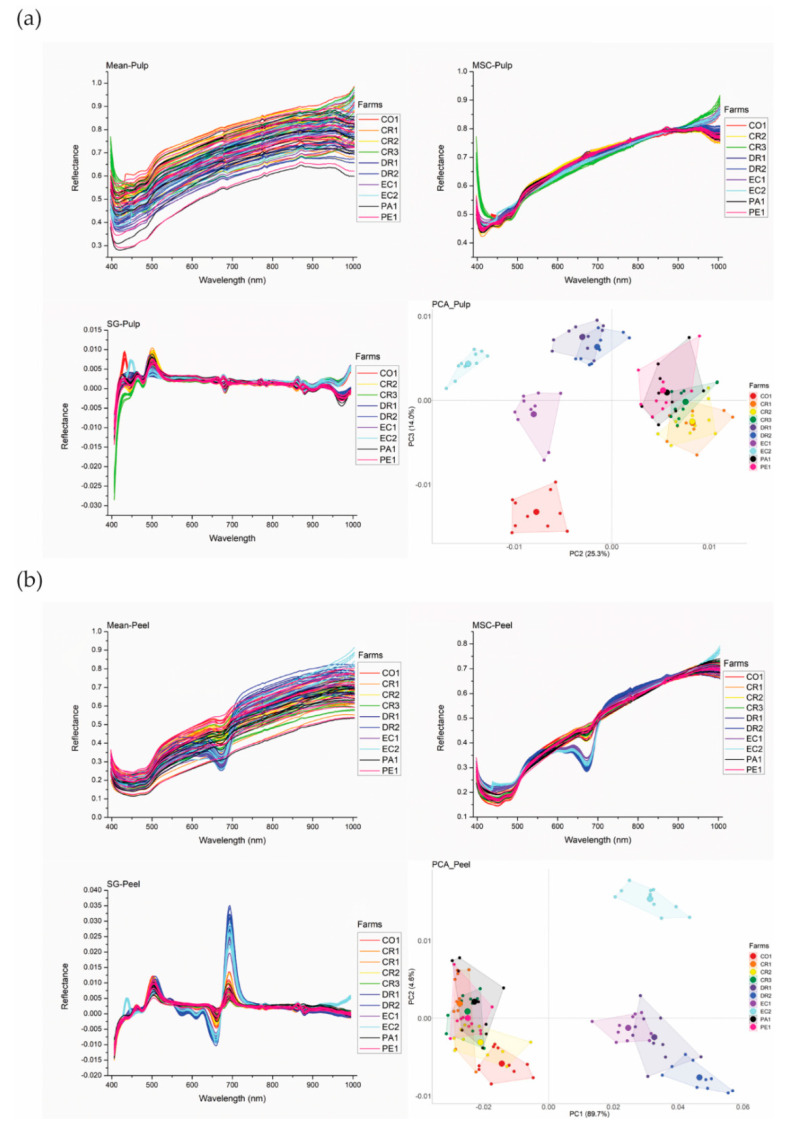
The mean reflectance (raw spectra), multiplicative scatter correction (MSC), Savitzky–Golay (SG) transformation spectra and principal component analysis (PCA) plot (based on Hyperspectral Imaging data) of banana pulp (**a**) and peel (**b**) samples from different countries (CO: Colombia; CR: Costa Rica; DR: Dominican Republic; EC: Ecuador; PA: Panama; PE: Peru; (*n* = 10 for each farm)). (Please refer to the web version of this article for the interpretation of the colours used in the figure).

The spectral profiles obtained from the hyperspectral images of banana pulp and peel samples are shown in Figure 3. Most of the samples show similar reflectance values in Mean and MSC spectra. Two necessary spectral preprocessing methods (e.g., MSC and SG transformation) were used to remove certain patterns and noise among variables and to further explore spectral differences. Particularly, SG can correct the spectrum baseline intensity caused by different particle sizes [30]. The transformed spectra of pulp and peel are shown in Figure 3. The SG transformation showed considerably more variation in spectra to reflect the spectral differences of pulp and peel samples. Furthermore, PCA was used to explore the grouping of banana pulp and peel samples from different farms based on their spectral signatures after SG transformation. The groups in the PCA plots are labelled at farm level other than the country level as several farms originated from the same countries but were cultivated with different production systems (i.e., organic and conventional methods). Therefore, the sample grouping based on geographical origin and production system can be seen in the same PCA.

The banana pulp from different farms and production systems shared similar patterns of mean spectra (Figure 3a). However, there were differences in the position and intensity of the absorption peak. The pulp spectra had absorption peaks in the following ranges: 400–500, 640–700 and 850–920 nm. The same is evident for the peel spectra. As shown in Figure 3b, the average spectra of the peel samples in the ranges 420–490 nm and 630–720 nm reflected obvious absorption peaks. The spectral differences could be caused by different geographical origins and growing conditions. For example, Sun et al. [17] reported that all rice samples from four different regions showed characteristic absorption peaks in ranges of approximately 500–1000 nm. However, another side to consider is the evidence, as reported in recent papers, that growing conditions normally influence and cause differences in the compositional traits of foods [5]. Therefore, it is also of value to look further into the effects of growing conditions, which is specific per geographical origin, on the physicochemical composition of bananas. For the current study, the growing conditions of the banana farms are summarised in Table 1. Table 2 shows the chemical composition of the banana samples, including moisture, total starch, total dietary fibre (TDF), protein, β-carotene contents and CIE L*a*b* values.

**Figure A1:**
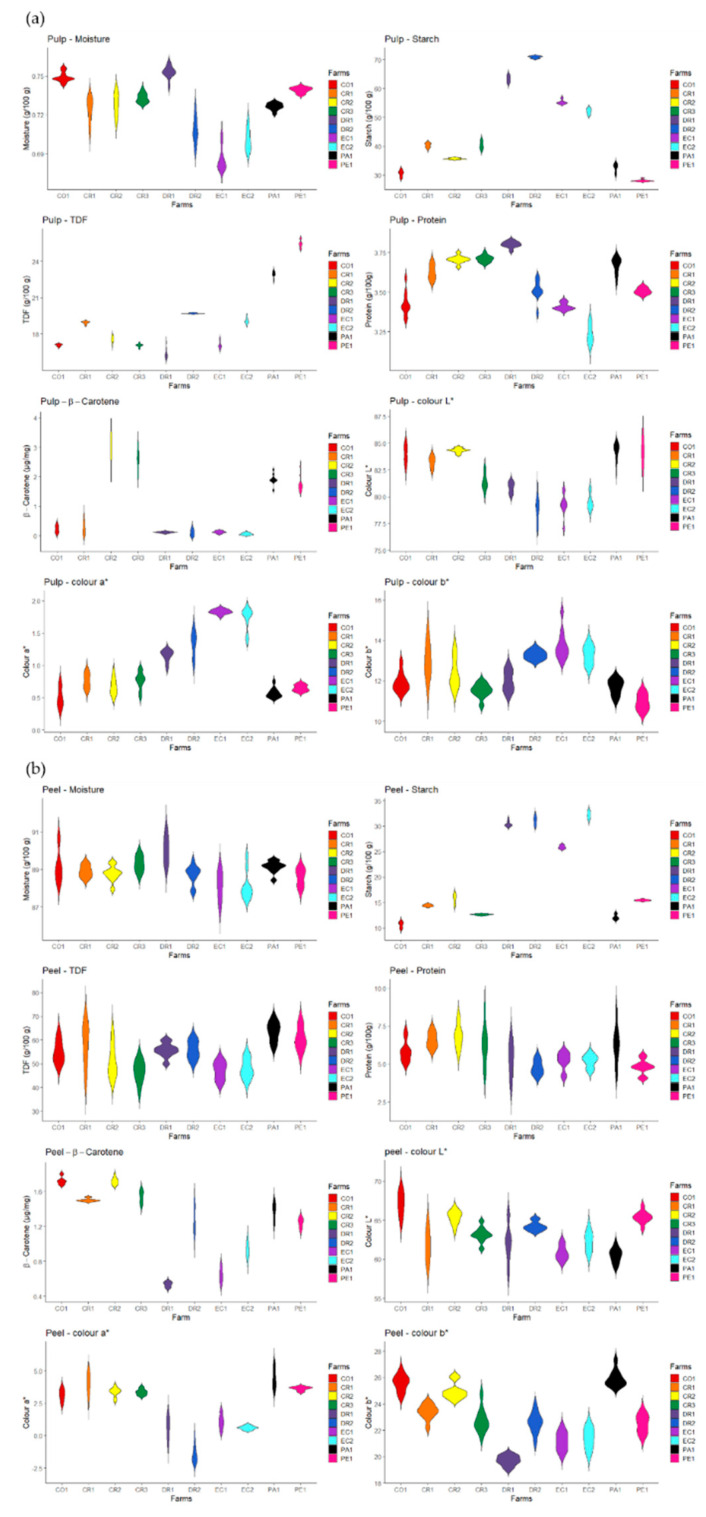
The violin plots of to show the data distributions of chemical compositions and colour values of banana pulp (**a**) and peel (**b**). (Please refer to the web version of this article for the interpretation of the colours used in the figure).

The data distribution of chemical and colour values were shown using violin plots in Figure A1. In brief, the chemical compositions and colour values of pulp and peel samples were different according to sampling sites. The differences could be conducted the variation of local growing conditions. It is has been reported that bananas harvested from a high altitude (300 m) usually have more aroma compounds and present a firmer texture than those harvested from a lower altitude (50 m) [31]. The highest altitude (726 m) was observed in one of the farms (CR1) in Costa Rica. The lowest altitude (10 m) was reported for farm PA1 from Panama (Table 1). Correspondingly, the chemical analysis indicated that banana pulp from CR1 had significantly higher total fibre content than PA1 (Table 2). The different absorptions at 876 nm and 967 nm in the NIR region of the banana pulp samples are related to the C–H group of fibre and the O–H stretching vibration of starch. Absorptions at 871, 967 and 972 nm typically represent the dietary fibre (C–H), starch (O–H) and saccharide (O–H) components in 0070eel, respectively [32]. Figure 2 shows the absorption differences in SG transformation spectra of pulp and peel in the above-mentioned wavelengths, which is linked to the starch and total dietary fibre contents (TDF) of the samples. Banana pulp and peel contained high amounts of starch and TDF (Table 2). For all the peel samples, the TDF contents are significantly higher than that of the pulp samples. This was expected as the peel is the outer, fibrous layer of the fruit, while the softer/less fibrous part is the pulp. In the current study, the total starch content of banana pulp ranged from 28.8 ± 4.4 to 70.9 ± 0.4 per 100 g DW (dry weight). When compared to the other farms, the banana pulp from PE1 had the lowest starch content at 28.8 ± 4.4% and also a lower rainfall (200 mm/year). While for TDF, the peel samples from the PA1 farm had the highest TDF content (63.6 ± 6.0%) with a higher rainfall of 3679.3 mm/year. For CO1, DR1, EC2, their TDF contents were significantly lower than PA1. These TDF findings are consistent with recent research on the composition of bananas [33,34]. These differences in composition could be due to rainfall as water is critical for the normal growth of bananas.

The values of the CIE L*a*b* colour coordinates were presented as mean ± standard deviation in Table 2 and Table 3. Higher b* values correspond to samples that appear more yellow, whereas with lower a* values the samples will appear more green. Similarly, the L* is for the lightness from black (lower/negative values) to white (higher/positive values). For the colour measurement of banana pulp and peel, the L* values and b* values showed a significant difference between most farms. In the visible spectral regions (350–780 nm) of banana pulp, wavelengths of 400–500 nm and 660–705 nm showed significant absorptions related to green and orange colours, which are consistent with the banana pulp colour values of ‘light yellow’ on the basis of the colour meter (b* value). Absorption at 455 nm and 460 nm indicates the presence of the carotenoids [35]. As reported, bananas with yellow and orange pulp were rich in β-carotene [36]. As found in Table 2, the banana pulp samples from Farm CR2 and CR3 show increased absorption at 455 nm and 460 nm, will likely be rich in β-carotene.

In Table 2 and Table 3, the content of β-carotene for pulp and peel showed significant differences between different countries, while the HSI spectral results were in line with these differences. For instance, the peel samples showed strong absorption in the 470–510 nm range, reflecting the green part of the peel, while absorption at 700 nm indicates a yellow-orange colour (b* value) for the peel. The variation in β-carotene content could be due to the growing conditions. As seen in Table 1, PA1 had the lowest monthly average temperature (19.7 °C) in terms of the average temperature of all sampling farms, whereas the highest (26.7 °C) was reported for DR1 and DR2 from the Dominican republic. A similar trend is seen for the β-carotene content of these samples, where banana pulp from PA1 had a higher β-carotene content (1.8 ± 0.2 μg/mg) than that of DR1 and DR2 (0.1 ± 0.1 μg/mg, respectively). Yang et al. [37] found that high temperatures contribute to represses chlorophyll degradation and influences the colour of banana peels. Thus, this could indicate that different growing conditions could result in differences in β-carotene contents. However, it is important to note that temperature is not the only growing condition that can influence β-carotene content. For these farms, other conditions could likely have played a role. This was consistent with other studies about fruits, which indicated that temperatures, altitudes and rainfall are correlated with secondary metabolite synthesis such as β-carotene [38,39]. Therefore, higher β-carotene content in CR2, CR3 could be caused by higher rainfall. Meantime, the high β-carotene content of PE1 could be due to organic production used in Peru, Bunea et al. [40] reported that organic agriculture resulted in higher ß-carotene concentration in grape. However, there are also papers illustrating no evidence of nutritional superiority of the organically grown fruits [38]. Yet, for the current study, there is not enough evidence to prove this effect and more research is needed for its verification. As for the β-carotene content in the peel samples, Colombia, Costa Rica, Peru and Panama had significantly higher contents compared to those from the Dominican Republic and Ecuador (Table 3). The content differences in β-carotene according to geographical origins were similar to results reported for other food products, such as acerola cherry [38], jujube fruit [39] and milk [41].

Differences in spectra due to the production system and growing conditions were also evident in the PCA plots (Figure 3). DR1 and DR2 were grouped separately from Peru (PE1), under the same organic production system, in the PCA plots of the pulp and peel samples (Figure 3). This grouping could be driven by the differences in altitude, temperature, rainfall and production systems that led to a compositional variation of starch and TDF contents which results in differences in the characteristic spectral values. The two farms from Ecuador, EC1 (organic) and EC2 (conventional), also practiced different ways of cultivation, and grouped separately in the PCA plot of the pulp and peel spectra (Figure 3); further indicating that the production system could potentially be indicated by HSI. In light of this, Su and Sun [42] found that the HSI conducted with PCA could easily separate organically and conventionally planted potatoes. Table 2 shows that EC1 and EC2 pulp samples had significant differences in TDF content. At the same time, the difference in rainfall of these two farms was also quite notably different; EC1 with 1511 mm/year and EC 2 with 843 mm/year.

A markable absorption around 995 nm that is likely related to the aromatic amines in pulp and peel spectral was identified, indicating that bananas often contain volatile substances and unique flavours [43]. For other compositional traits such as moisture and protein content, it is easy to recognise that banana peel has more moisture than pulp (~89% vs. ~74%, respectively) because the total starch content accounts for a large proportion of banana pulp. The pulp samples from DR1 had the highest moisture content (75.3 ± 5.9%), which is significantly higher than banana pulp samples from CR1, EC1 and EC2. Nguyen et al. [44] found the moisture content (on a wet basis) to be 74.7 ± 1.3% for banana pulp from Australia, which reflects the similar values of the current study. For the moisture content of peel samples, the samples from the Dominican Republic (DR2) had the highest value, while the lowest content was reported in Ecuador (EC2). However, there are no significant differences between different groups, which means growing conditions did not have a great influence on the moisture content of bananas. Lei et al. [45] reported that bending vibration around 700 nm (N–H) was attributed to protein. In Figure 3, the variation of HSI spectra around 700 nm could be caused by different protein contents of pulp and peel. Generally, the banana peel samples have higher protein contents than the pulp samples (~5–6% vs. ~3–4%, respectively). PCA plots also showed that in comparison with the protein content of the pulp samples, the results revealed that banana peel had higher protein contents, such as 6.8 ± 0.7% for CR2. However, only a few farms showed significant differences in pulp and peel samples concerning the protein contents from all sampling countries. The DR1 bananas had considerably higher protein contents than bananas from other farms for pulp and reflected remarkable differences comparing peel samples from farm CR2 (*p* < 0.05). Table 1 showed that DR1 had quite a higher altitude, which could be the underlying mechanism. Mohapatra et al. also reported that the different content of banana protein could be caused by genomic mutation, altitude and climate [46].

Not only the effects of geographical factors but also the effects of production systems were reflected in PCA plots. As seen in Figure 2, three organic farms (DR1, DR2 and EC1), were separated from other conventional farms both based on HSI spectra of pulp and peel, which proved that the effects of different production systems can be observed in the hyperspectral results. In further, compositional traits from Table 2 also showed that pulp from organic cultivation had more contents of Moisture, starch and total dietary fibre and less β-carotene significantly (*p* < 0.05) in comparison with conventional methods. The notably higher starch content also could be found in peel samples (*p* < 0.05). However, one of the organic farms, namely PE1, can not be separated from other conventional farms. This suggested that the difference in the HSI spectrum is due to the combined action of growing conditions. Lima et al. [47] also pointed out that organic agriculture has great potential to increase the content of certain nutrients in food, but, obviously, there are more factors such as crop time, climate, soil characteristics, environmental conditions still make contributions.

Previous studies usually used the PCA plots to show the grouping of samples based on their different geographic locations [5,28]. However, our research shows that the potential differences in spectra are not only caused by geographical origin, but are also very much related to the characteristic growth conditions such as altitude, temperature and rainfall. Even for the bananas collected from the neighboring countries such as Colombia and Panama, the PCA plots showed that CO1 grouped separately from PA1 according to the pulps’ spectral fingerprint as both farms have large differences in altitude and rainfall. For the peel samples, although the DR1 and EC1 farms had a quite long geographical distance between them, they were still grouped closely. This could be because both of them shared similar temperature and rainfall, and were cultivated with a similar organic system. In view of this, Sun et al. [35] also demonstrated that HSI conducted with PCA could address the difference between two shrimp groups from high- and low-salinity environments.

### 3.2. Correlation of the HSI Spectra, Compositional Traits Colour Data and Growing Conditions

**Figure 4:**
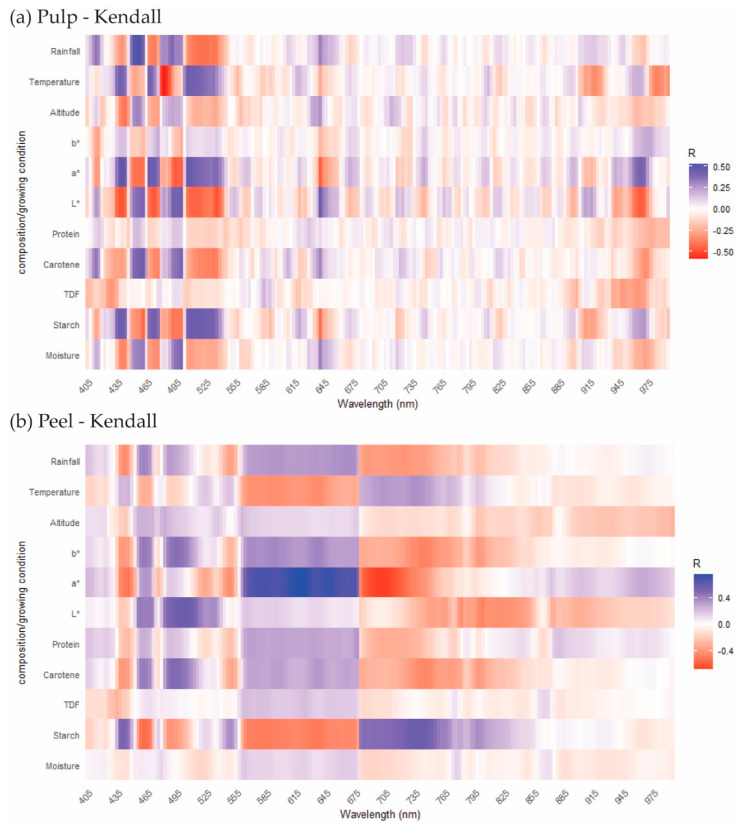
The heatmap to show the Kendall correlations (r) between wavelength, composition and growing condition of banana pulp (**a**) and peel (**b**). (Please refer to the web version of this article for the interpretation of the colours used in the figure).

In the last ten years, HSI technology has been widely used in food authenticity and fraud research, especially adulteration, agricultural product traceability, organic food identification and other related fields [48]. However, most articles mainly tend to use hyperspectral techniques to accurately identify sample differences, but few works of literature report the causes of these differences. In the above parts of this paper, the chemical compositions and value databases from banana pulp and peel samples were obtained by a series of approaches. The growing conditions and HSI spectral data were acquired as well. To explore how HSI spectra relate to the differences in compositional traits and growing conditions, the spectral data were correlated with the compositional data, colour values and growing conditions (Figure 4).

Figure 4a shows the correlation between the HSI spectra (Savitzky–Golay transformation) on the X axis and the compositional, colour, growing conditions on the Y axis. The correlation analysis was based on the characteristic peaks caused by certain compositions and colours of pulp and peel under HSI wavelengths 400–1000 nm [28]. For the wavelength ranges 405–525 nm, 615–645 nm and 885–985 nm, a relatively strong correlation was observed. The HSI spectra of the banana pulp samples (Figure 4a) also showed higher differences in these ranges according to different growing conditions. Compared with protein, TDF, moisture, the carotene and starch contents showed a stronger correlation with HSI spectra in the 405–525 nm and 615–625 nm wavelength ranges. As the most abundant component of banana pulp, the moderate positive correlation (r = 0.39–0.45, *p* < 0.05) of starch with HSI indicates the potential of HSI to identify starch-rich foods. As reported in Section 3.1, absorptions at 876 nm and 967 nm are generally related to C–H group of fibre and O–H stretching vibration of starch [49], therefore moderate correlations (r values around 0.40, *p* < 0.05) of starch of pulp also were shown in the 885–985 nm range. Compared to banana pulp, weaker correlations of most of the traits and HSI exist for the peel sample. This is possibly due to the fact that banana pulp is more likely to reflect the influence of growing conditions on the composition and colour values. At the same time, the spectra of the banana peel and the PCA results also showed that the difference between the banana peels is smaller than those for the pulps. Ultimately, HSI could reveal the difference in the composition of bananas and is a suitable tool to link the compositional traits to the growing conditions of bananas.

### 3.3. Correlation between Growing Conditions and Banana Composition

**Figure 5:**
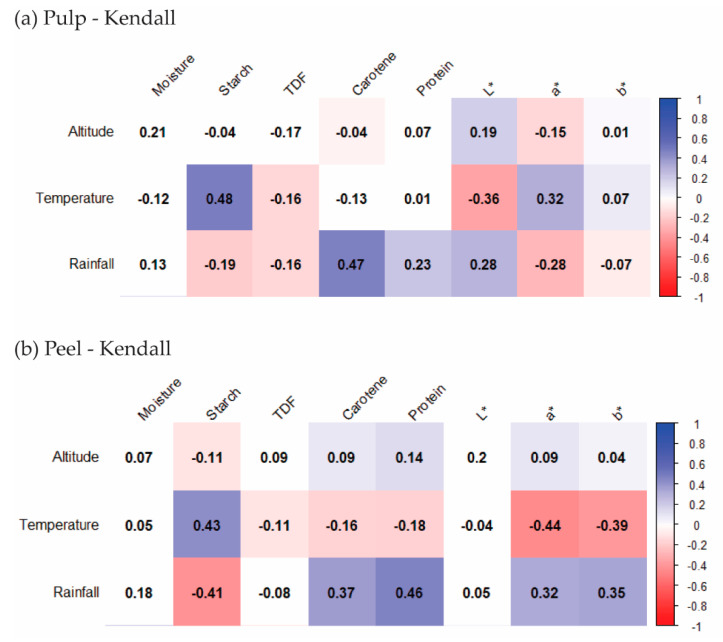
The heatmap to show the Kendall correlation (r) between growing condition and composition traits of banana pulp (**a**) and peel (**b**). (please refer to the web version of this article for the interpretation of the colours used in the figure.).

Research studies on bananas have mainly reported the relationships between the climate and yield of bananas, and few papers have focused on the correlation of local growing conditions and banana compositional traits [9]. The contents of chemical compositions of plants the results of absorption, utilization and metabolic activities [50]. The starch content of sweet potatoes was increased in high altitudes farms [51]. The effects of different altitudes and temperatures on moisture content, reducing sugars, crude fibre as well as color were studied in *yacon tuberous* roots [52]. However, the correlations of growing conditions and food compositions were still scarcely reported in the literature. To understand the effects of growing conditions on the composition and colour variation of the bananas, correlations between those datasets were investigated by Kendall’s correlation coefficients (r). In Figure 5, The coefficients and the significant correlations are shown from red (negative) to blue (positive) (*p* < 0.05), while the insignificant correlations are marked as blank. There are few publications reporting on correlations between the growing condition of banana and the composition. It was reported that the harvest season of the banana field will affect the firmness of the banana fruit [10]. Apart from this, some research studies reported that a quite strong correlation (r = 0.62–0.94) was noted between rainfall levels and fruit firmness [53]. In the review, the cited research showed that the relationship of climate differences and fruit quality, the vitamin content, phenolic compound content and micronutrients of fruits are highly affected by temperature change, water availability and other geographical variables [9].

For the pulp samples, mostly positive correlations were found between temperature, rainfall, chemical composition and colour values (Figure 5a). The colour index a* had a moderate and slightly positive correlation with temperature (r = 0.32, *p* < 0.05), while the L* factor correlated negatively with temperature. For the former, the positive correlation means that with increasing temperature an increase in a* values (more red and less green colour) can be expected. This finding coincides with the fact that a higher temperature is helpful to the growth of bananas and promotes the pulp to change from white to light yellow. This also explains why the L* value is positively correlated with temperature. Because L* stands for lightness of samples, the lightness of pulp will be decreased in the colour transformation during growth. Growing conditions such as rainfall resulted in a moderate positive correlation (r = 0.47, *p* < 0.05) with carotene content for the banana pulp. Therefore, higher carotene contents can be expected for bananas grown in regions with more extensive rainfall. In our research, the banana pulp from CR2 had the highest carotene content, also the highest annual rainfall is reported on this farm as well. Most of the chemical and colour values were only slightly influenced by altitude, indicating that the effects of temperature and rainfall were higher than those of altitude.

In contrast to the pulp samples, temperature largely showed significant negative correlations with most of the chemical components and colour values of the peel samples. The temperature had a slight negative correlation with the protein content of peel samples (r = −0.18, *p* < 0.05), which could be due to the effect of temperature on banana metabolism. Similar correlations were determined for the protein contents of salmon fillets. Ørnholt-Johansson et al. [49] reported that a negative relationship exists between the protein content of salmon and sea temperature.

Particularly, the temperature had a negative correlation with carotene content (r = −0.16, *p* < 0.05), which is consistent with the findings in carrot research [54]. As we found in Section 3.1, the banana pulp farm PA1 within high temperature showed higher β-carotene content than that of DR1 and DR2 within lower temperatures. A similar study about the influences of temperature on bananas colour also reported that high temperature could decrease the yellow colour of bananas [37]. A negative correlation between temperature and b* value (r = −0.39, *p* < 0.05) was observed, whereas the b* value reflects the yellow colour of banana peel. A moderate positive correlation was observed between rainfall and the b* colour value (r = 0.35, *p* < 0.05); hence, higher rainfall could enhance the banana peel colour as an increase in b* value will result in a more intense yellow colour of the peel. As reported in Section 3.1, farm PE1 with the lowest rainfall showed the lowest b* value compared with other farms (*p* < 0.05). From Figure 5b, the same correlation trends were observed between the results of carotene content and colour indexes of peel samples. The impact of growing conditions on the fruit’s compositions is due to the effect of temperature and rainfall affecting the metabolism of nutrients in fruits [55].

These results demonstrate that different growing conditions had certain effects on the chemical compositions and colour values of banana pulp and peel samples. Climatic factors, such as annual average precipitation, relative humidity and average temperature, have also been reported to have either positive or negative effects on the metabolites of *Flos Carthami* flowers [56]. The different altitudes were also reported having influences on organic acids and aroma volatile attributes of pomegranate fruit [57]. Similarly, CIE L*a*b* colour spaces are useful for the identification of honey types from different geographical locations [58]. When discussing food authenticity, it is vital to consider the growing conditions as food quality, flavour and economic value often benefit from special geographical conditions.

## 4. Conclusions

The results demonstrate that banana pulp and peel from different geographical origins and production systems have unique spectral fingerprints that can be linked to their chemical composition and associated growing conditions. PCA revealed that the spectral range from 400–1000 nm is an effective range to show the differences in geographical origin and production system of bananas using a portable HSI system. The combined effects of rainfall, temperature and altitude are likely some of the main causes for the variation in the carotene, starch and dietary fibre contents. The colour values are more related to altitude and temperature at the geographical locations. The wavelength ranges 405–525 nm, 615–645 nm and 885–985 nm of HSI were significantly correlated (r values around ± 0.50, *p* < 0.05) with the carotene content, starch content, and a* and b* colour values. Indirectly, the differences in HSI spectral data could reflect the effects of rainfall, temperatures and altitudes (growing conditions) on compositional traits of bananas. These findings help to better understand the effects of growing conditions on the colour differences and the variation in the composition of bananas.

## Figures and Tables

**Table 1:** The number of banana samples (pulp and peel) collected per country as well as the associated growing conditions.

Country	Farm Code	Pulp	Peel	Production System	Altitude (m)	Monthly Mean Temperature (°C)	Annual Rainfall (mm/year)
Colombia	CO1	10	10	Conventional	66	23.2	1837
Costa Rica	CR1	10	10	Conventional	726	23.4	2857
	CR2	10	10	Conventional	47	24.4	5014
	CR3	10	10	Conventional	24	26.3	4378
Dominica Republic	DR1	10	10	Organic	65	26.7	925
	DR2	10	10	Organic	27	26.7	925
Ecuador	EC1	10	10	Organic	32	22.9	1511
	EC2	10	10	Conventional	22	26.5	843
Panama	PA1	10	10	Conventional	10	19.7	3679
Peru	PE1	10	10	Organic	40	24.1	200

**Table 2:** The composition of banana pulp samples for the different countries (*n* = 10 for each farm).

Country	Farm Code	Production System	Moisture ^#^ (g/100 g)	Starch (g/100 g)	Total Dietary Fibre (g/100 g)	Protein (g/100 g)	β-Carotene (μg/mg)	L*	a*	b*
Colombia	CO1	Conventional	74.8 ^ab^ ± 1.7	31.4 ^d^ ± 5.5	17.6 ^bd^ ± 1.8	3.4 ^ab^ ± 0.3	0.2 ^d^ ± 0.1	83.8 ^a^ ± 0.6	0.4 ^b^ ± 0.2	12.1 ^a^ ± 0.5
Costa Rica	CR1	Conventional	72.4 ^bc^ ± 0.8	41.0 ^cd^ ± 4.1	19.4 ^bc^ ± 1.9	3.6 ^ab^ ± 0.3	0.2 ^d^ ± 0.2	83.5 ^a^ ± 0.7	0.7 ^ab^ ± 0.1	13.1 ^b^ ± 0.8
	CR2	Conventional	72.8 ^ac^ ± 0.7	36.4 ^d^ ± 1.5	17.5 ^bd^ ± 2.7	3.7 ^ab^ ± 0.3	3.0 ^a^ ± 0.4	84.1 ^a^ ± 0.4	0.7 ^b^ ± 0.1	12.5 ^a^ ± 0.8
	CR3	Conventional	73.0 ^ac^ ± 1.0	40.1 ^cd^ ± 5.2	17.0 ^cd^ ± 1.2	3.7 ^ab^ ± 0.2	2.5 ^b^ ± 0.3	81.5 ^b^ ± 0.8	0.7 ^b^ ± 0.1	11.4 ^b^ ± 0.4
Dominica Republic	DR1	Organic	75.3 ^a^ ± 5.9	62.0 ^ab^ ± 4.5	16.2 ^d^ ± 1.2	3.8 ^a^ ± 0.5	0.1 ^d^ ± 0.0	80.8 ^bc^ ± 0.7	1.1 ^c^ ± 0.1	11.8 ^d^ ± 0.5
	DR2	Organic	70.6 ^cd^ ± 1.0	70.9 ^a^ ± 0.4	19.7 ^b^ ± 1.75	3.5 ^ab^ ± 0.4	0.1 ^d^ ± 0.0	78.9 ^d^ ± 1.8	1.3 ^d^ ± 0.2	13.2 ^b^ ± 0.2
Ecuador	EC1	Organic	68.8 ^d^ ± 1.0	55.4 ^b^ ± 6.8	17.0 ^cd^ ± 2.0	3.4 ^ab^ ± 0.7	0.1 ^d^ ± 0.1	79.3 ^d^ ± 1.1	1.8 ^c^ ± 0.2	14.1 ^c^ ± 0.7
	EC2	Conventional	69.4 ^d^ ± 1.7	52.1 ^bc^ ± 6.2	19.1 ^bc^ ± 2.4	3.2 ^ab^ ± 0.1	0.0 ^d^ ± 0.0	79.7 ^cd^ ± 0.9	1.6 ^c^ ± 0.3	13.1 ^c^ ± 0.5
Panama	PA1	Conventional	72.6 ^ac^ ± 0.3	33.4 ^d^ ± 2.8	22.9 ^a^ ± 1.5	3.7 ^ab^ ± 0.3	1.8 ^c^ ± 0.2	84.1 ^a^ ± 0.8	0.5 ^a^ ± 0.1	11.7 ^a^ ± 0.3
Peru	PE1	Organic	74.0 ^ab^ ± 0.4	28.8 ^d^ ± 4.4	25.5 ^a^ ± 1.3	3.5 ^ab^ ±0.2	1.9 ^c^ ± 0.5	84.1 ^a^ ± 1.2	0.6 ^ab^ ± 0.2	10.9 ^b^ ± 0.3

^a–d^ Different mean values in the same column with different superscript letters are significantly different (*p* < 0.05) according to Tukey’s significant difference test; ^#^ Moisture content is based on wet weight, other compositions are based on freeze-dried (dry) weight.

**Table 3:** The composition of banana peel samples for the different countries (*n* = 10 for each farm).

Country	Farm Code	Production System	Moisture ^#^ (g/100 g)	Starch (g/100 g)	Total Dietary Fibre (g/100 g)	Protein (g/100 g)	β-Carotene (μg/mg)	L*	a*	b*
Colombia	CO1	Conventional	89.2 ^bd^ ± 0.7	10.9 ^c^ ± 2.6	55.0 ^bcd^ ± 4.7	5.8 ^bd^ ± 0.7	1.7 ^a^ ± 0.1	67.1 ^a^ ± 1.4	3.2 ^b^ ± 0.5	25.7 ^a^ ± 0.7
Costa Rica	CR1	Conventional	88.9 ^cd^ ± 0.3	14.5 ^c^ ± 1.9	58.1 ^ac^ ± 8.9	6.6 ^b^ ± 0.5	1.5 ^ac^ ± 0.5	62.7 ^bce^ ± 3.6	3.9 ^ab^ ± 0.7	23.2 ^b^ ± 0.6
	CR2	Conventional	88.6 ^cd^ ± 0.9	15.7 ^c^ ± 1.0	51.1 ^ce^ ± 6.8	6.8 ^b^ ± 0.7	1.7 ^a^ ± 0.1	65.6 ^ab^ ± 1.0	3.4 ^b^ ± 0.3	24.8 ^a^ ± 0.6
	CR3	Conventional	89.5 ^bc^ ± 0.9	12.6 ^c^ ± 0.1	46.9 ^ce^ ± 4.5	6.2 ^bc^ ± 1.2	1.5 ^ac^ ± 0.1	63.8 ^bcd^ ± 1.7	3.4 ^b^ ± 0.2	23.0 ^b^ ± 1.1
Dominica Republic	DR1	Organic	90.4 ^b^ ± 2.0	30.3 ^ab^ ± 1.9	55.8 ^bc^ ± 2.9	5.3 ^cd^ ± 1.1	0.5 ^c^ ± 0.1	62.0 ^ce^ ± 2.2	0.6 ^c^ ± 0.7	19.6 ^c^ ± 1.2
	DR2	Organic	88.4 ^cd^ ± 0.8	31.7 ^a^ ± 1.7	55.9 ^bc^ ± 8.7	4.7 ^d^ ± 0.4	1.2 ^ac^ ± 0.2	64.4 ^ac^ ± 1.7	−1.5 ^d^ ± 0.6	22.6 ^b^ ± 0.6
Ecuador	EC1	Organic	88.1 ^d^ ± 0.6	25.6 ^b^ ± 3.2	47.1 ^ce^ ± 6.7	5.2 ^cd^ ± 0.5	0.6 ^c^ ± 0.1	60.9 ^de^ ± 0.8	1.1 ^c^ ± 0.5	21.1 ^c^ ± 0.6
	EC2	Conventional	88.0 ^d^ ± 0.6	32.7 ^a^ ± 1.9	47.9 ^de^ ± 9.0	5.1 ^cd^ ± 0.4	0.9 ^c^ ± 0.1	62.3 ^ce^ ± 1.1	0.7 ^c^ ± 0.5	21.3 ^c^ ± 0.7
Panama	PA1	Conventional	89.1 ^bd^ ± 0.3	12.8 ^c^ ± 1.8	63.6 ^a^ ± 6.0	6.1 ^bc^ ± 0.7	1.4 ^ac^ ± 0.3	60.1 ^e^ ± 5.4	4.6 ^a^ ± 0.7	25.0 ^a^ ± 1.6
Peru	PE1	Organic	88.5 ^cd^ ± 0.4	15.5 ^c^ ± 0.2	60.7 ^ab^ ± 7.8	4.8 ^d^ ± 0.4	1.2 ^b^ ± 0.1	65.0 ^ac^ ± 2.6	3.9 ^ab^ ± 0.7	22.9 ^c^ ± 1.5

^a–e^ Different mean values in the same column with different superscript letters are significantly different (*p* < 0.05) according to Tukey’s significant difference test; ^#^ Moisture content is based on wet weight, other compositions are based on freeze-dried (dry) weight.

## References

[1] Pereira A., Maraschin M. (2015). Banana (*Musa* spp.) from peel to pulp: Ethnopharmacology, source of bioactive compounds and its relevance for human health. J. Ethnopharmacol..

[2] Singh B., Singh J.P., Kaur A., Singh N. (2016). Bioactive compounds in banana and their associated health benefits—A review. Food Chem..

[3] Sulaiman S.F., Yusoff N.A.M., Eldeen I.M., Seow E.M., Sajak A.A.B., Ooi K.L. (2011). Correlation between total phenolic and mineral contents with antioxidant activity of eight Malaysian bananas (*Musa* sp.). J. Food Compos. Anal..

[4] Aurore G., Parfait B., Fahrasmane L. (2009). Bananas, raw materials for making processed food products. Trends Food Sci. Technol..

[5] Margraf T., Santos É.N.T., de Andrade E.F., van Ruth S.M., Granato D. (2016). Effects of geographical origin, variety and farming system on the chemical markers and in vitro antioxidant capacity of Brazilian purple grape juices. Food Res. Int..

[6] Ben Ghorbal A., Leventdurur S., Agirman B., Boyaci Gunduz C.P., Kelebek H., Carsanba E., Darici M., Erten H. (2018). Influence of geographic origin on agronomic traits and phenolic content of cv. Gemlik olive fruits. J. Food Compos. Anal..

[7] Li X., Wasila H., Liu L., Yuan T., Gao Z., Zhao B., Ahmad I. (2015). Physicochemical characteristics, polyphenol compositions and antioxidant potential of pomegranate juices from 10 Chinese cultivars and the environmental factors analysis. Food Chem..

[8] Drappier J., Thibon C., Rabot A., Geny Denis L. (2019). Relationship between wine composition and temperature: Impact on Bordeaux wine typicity in the context of global warming—Review. Crit. Rev. Food Sci. Nutr..

[9] Stewart A.L., Ahmed S., Srivastava A.K., Hu C.B.T.F.C. (2020). Effects of climate change on fruit nutrition. Fruit Crops.

[10] Fu X., Cheng S., Liao Y., Huang B., Du B., Zeng W., Jiang Y., Duan X., Yang Z. (2018). Comparative analysis of pigments in red and yellow banana fruit. Food Chem..

[11] FAO (2019). Banana Market. Review: Preliminary Results for 2018.

[12] Medina S., Perestrelo R., Silva P., Pereira J.A.M., Câmara J.S. (2019). Current trends and recent advances on food authenticity technologies and chemometric approaches. Trends Food Sci. Technol..

[13] Li L., Xie S., Ning J., Chen Q., Zhang Z. (2019). Evaluating green tea quality based on multisensor data fusion combining hyperspectral imaging and olfactory visualization systems. J. Sci. Food Agric..

[14] Xu J., Gowen A.A. (2019). Spatial-spectral analysis method using texture features combined with PCA for information extraction in hyperspectral images. J. Chemom..

[15] Oliveira M.M., Cruz Tirado J.P., Barbin D.F. (2019). Nontargeted analytical methods as a powerful tool for the authentication of spices and herbs: A review. Compr. Rev. Food Sci. Food Saf..

[16] Acierno V., Fasciani G., Kiani S., Caligiani A., van Ruth S. (2019). PTR-QiToF-MS and HSI for the characterization of fermented cocoa beans from different origins. Food Chem..

[17] Sun J., Lu X., Mao H., Jin X., Wu X. (2017). A Method for rapid identification of rice origin by hyperspectral imaging technology. J. Food Process. Eng..

[18] Choi J.Y., Heo S., Bae S., Kim J., Moon K.D. (2020). Discriminating the origin of basil seeds (*Ocimum basilicum* L.) using hyperspectral imaging analysis. LWT.

[19] Mditshwa A., Magwaza L.S., Tesfay S.Z., Mbili N. (2017). Postharvest quality and composition of organically and conventionally produced fruits: A review. Sci. Hortic..

[20] Latin America Map Free Templates. www.Yourfreetemplates.com/.

[21] Harris I.C., Jones P.D., Osborn T. (2020): CRU TS4.04: Climatic Research Unit (CRU) Time-Series (TS) version 4.04 of high-resolution gridded data of month-by-month variation in climate (Jan. 1901- Dec. 2019). Centre for Environmental Data Analysis. https://catalogue.ceda.ac.uk/uuid/89e1e34ec3554dc98594a5732622bce9.

[22] Behmann J., Acebron K., Emin D., Bennertz S., Matsubara S., Thomas S., Bohnenkamp D., Kuska M., Jussila J., Salo H. (2018). Specim IQ: Evaluation of a new, miniaturized handheld hyperspectral camera and its application for plant phenotyping and disease detection. Sensors.

[23] Park B., Lu R., Park B., Lu R. (2015). Hyperspectral Imaging Technology in Food and Agriculture.

[24] Srichuwong S., Curti D., Austin S., King R., Lamothe L., Gloria Hernandez H. (2017). Physicochemical properties and starch digestibility of whole grain sorghums, millet, quinoa and amaranth flours, as affected by starch and non-starch constituents. Food Chem..

[25] Jung S., Rickert D.A., Deak N.A., Aldin E.D., Recknor J., Johnson L.A., Murphy P.A. (2003). Comparison of kjeldahl and dumas methods for determining protein contents of soybean products. J. Am. Oil Chem. Soc..

[26] Ayustaningwarno F., van Ginkel E., Vitorino J., Dekker M., Fogliano V., Verkerk R. (2020). Nutritional and physicochemical quality of vacuum-fried mango chips is affected by ripening stage, frying temperature, and time. Front. Nutr..

[27] Ashwar B.A., Gani A., Wani I.A., Shah A., Masoodi F.A., Saxena D.C. (2016). Production of resistant starch from rice by dual autoclaving-retrogradation treatment: Invitro digestibility, thermal and structural characterization. Food Hydrocoll..

[28] Yin W., Zhang C., Zhu H., Zhao Y., He Y. (2017). Application of near-infrared hyperspectral imaging to discriminate different geographical origins of Chinese wolfberries. PLoS ONE.

[29] Shapiro S.S., Wilk M.B. (1965). An Analysis of Variance Test for Normality (Complete Samples). Biometrika.

[30] Gao J., Li X., Zhu F., He Y. (2013). Application of hyperspectral imaging technology to discriminate different geographical origins of *Jatropha curcas* L. seeds. Comput. Electron. Agric..

[31] Bugaud C., Chillet M., Beauté M.P., Dubois C. (2006). Physicochemical analysis of mountain bananas from the French West Indies. Sci. Hortic..

[32] Workman J., Weyer L. (2007). Practical Guide to Interpretive Near-Infrared Spectroscopy.

[33] Emaga T.H., Bindelle J., Agneesens R., Buldgen A., Wathelet B., Paquot M. (2011). Ripening influences banana and plantain peels composition and energy content. Trop. Anim. Health Prod..

[34] Pareek S. (2015). Nutritional and Biochemical Composition of Banana (*Musa spp.*) Cultivars. Nutritional Composition of Fruit Cultivars.

[35] Sun D., Weng H., He X., Li L., He Y., Cen H. (2019). Combining near-infrared hyperspectral imaging with elemental and isotopic analysis to discriminate farm-raised pacific white shrimp from high-salinity and low-salinity environments. Food Chem..

[36] Bugaud C., Daribo M.O., Dubois C. (2007). Climatic conditions affect the texture and colour of Cavendish bananas (Grande Naine cultivar). Sci. Hortic..

[37] Yang X., Song J., Fillmore S., Pang X., Zhang Z. (2011). Effect of high temperature on color, chlorophyll fluorescence and volatile biosynthesis in green-ripe banana fruit. Postharvest Biol. Technol..

[38] Cardoso P.C., Tomazini A.P.B., Stringheta P.C., Ribeiro S.M.R., Pinheiro Sant’Ana H.M. (2011). Vitamin C and carotenoids in organic and conventional fruits grown in Brazil. Food Chem..

[39] Reche J., Hernández F., Almansa M.S., Carbonell-Barrachina Á.A., Legua P., Amorós A. (2019). Effects of organic and conventional farming on the physicochemical and functional properties of jujube fruit. LWT.

[40] Bunea C.I., Pop N., Babeş A.C., Matea C., Dulf F.V., Bunea A. (2012). Carotenoids, total polyphenols and antioxidant activity of grapes (*Vitis vinifera*) cultivated in organic and conventional systems. Chem. Cent. J..

[41] Brodziak A., Król J., Litwińczuk Z., Barłowska J. (2018). Differences in bioactive protein and vitamin status of milk from certified organic and conventional farms. Int. J. Dairy Technol..

[42] Su W., Sun D. (2016). Potential of hyperspectral imaging for visual authentication of sliced organic potatoes from potato and sweet potato tubers and rapid grading of the tubers according to moisture proportion. Comput. Electron. Agric..

[43] Boudhrioua N., Giampaoli P., Bonazzi C. (2003). Changes in aromatic components of banana during ripening and air-drying. LWT Food Sci. Technol..

[44] Nguyen M.H., Price W.E. (2007). Air-drying of banana: Influence of experimental parameters, slab thickness, banana maturity and harvesting season. J. Food Eng..

[45] Lei Y., Zhou Q., Zhang Y., Chen J., Sun S., Noda I. (2010). Analysis of crystallized lactose in milk powder by Fourier-transform infrared spectroscopy combined with two-dimensional correlation infrared spectroscopy. J. Mol. Struct..

[46] Mohapatra D., Mishra S., Sutar N. (2010). Banana and its by-product utilisation: An overview. J. Sci. Ind. Res..

[47] Lima G.P.P., Vianello F. (2011). Review on the main differences between organic and conventional plant-based foods. Int. J. Food Sci. Technol..

[48] Pu H., Lin L., Sun D. (2019). Principles of hyperspectral microscope imaging techniques and their applications in food quality and safety detection: A review. Compr. Rev. Food Sci. Food Saf..

[49] Ørnholt Johansson G., Frosch S., Munk Jørgensen B. (2017). Variation in some quality attributes of Atlantic salmon fillets from aquaculture related to geographic origin and water temperature. Aquaculture.

[50] Manach C., Scalbert A., Morand C., Rémésy C., Jiménez L. (2004). Polyphenols: Food sources and bioavailability. Am. J. Clin. Nutr..

[51] Tumwegamire S., Kapinga R., Rubaihayo P.R., LaBonte D.R., Grüneberg W.J., Burgos G., Felde T.Z., Carpio R., Pawelzik E., Mwanga R.O.M. (2011). Evaluation of Dry Matter, Protein, Starch, Sucrose, β-carotene, Iron, Zinc, Calcium, and Magnesium in East African Sweetpotato [*Ipomoea batatas* (L.) Lam] Germplasm. HortScience.

[52] Silva D.M.N., Lima R.R., Oliveira F.L., Teixeira L.J.Q., Machado L.C.A. (2018). Physical and chemical characterization of yacon tuberous roots at different altitudes and planting times. Hortic. Bras..

[53] de Jesús Ornelas-Paz J., Quintana Gallegos B.M., Escalante Minakata P., Reyes Hernández J., Pérez Martínez J.D., Rios Velasco C., Ruiz Cruz S. (2018). Relationship between the firmness of Golden Delicious apples and the physicochemical characteristics of the fruits and their pectin during development and ripening. J. Food Sci. Technol..

[54] Sulaeman A., Keeler L., Giraud D.W., Taylor S.L., Wehling R.L., Driskell J.A. (2001). Carotenoid content and physicochemical and sensory characteristics of carrot chips deep-fried in different oils at several temperatures. J. Food Sci..

[55] Sim I., Suh D.H., Singh D., Do S.G., Moon K.H., Lee J.H., Ku K.M., Lee C.H. (2017). Unraveling metabolic variation for blueberry and chokeberry cultivars harvested from different geo-climatic regions in Korea. J. Agric. Food Chem..

[56] Cao T., Liu J., Zhang X., Wei X., Qi Y., Zhang B., Liu H., Xiao P. (2019). Metabolomics characterization of different geographical origins of Flos Carthami using UPLC-QTOF/MS and their correlation with climate factors. Anal. Methods.

[57] Mphahlele R.R., Caleb O.J., Fawole O.A., Opara U.L. (2016). Effects of different maturity stages and growing locations on changes in chemical, biochemical and aroma volatile composition of ‘Wonderful’ pomegranate juice. J. Sci. Food Agric..

[58] Tuberoso C.I.G., Jerković I., Sarais G., Congiu F., Marijanović Z., Kuś P.M. (2014). Color evaluation of seventeen European unifloral honey types by means of spectrophotometrically determined CIE $L^{*}C_{\mathrm{ab}}^{*}h_{\mathrm{ab}}^{o}$ chromaticity coordinates. Food Chem..

